# Retraction: Wasp venom peptide improves the proapoptotic activity of alendronate sodium in A549 lung cancer cells

**DOI:** 10.1371/journal.pone.0330467

**Published:** 2025-08-18

**Authors:** The PLOS One Editors

Following the publication of this article [1], the following concerns were identified:

- The study relies on retracted work cited in References 27, 32, 43, 48, 49 and 56.
- The article includes multiple statements that do not appear to be supported by the cited references, or for which no references were provided.

In addition, the underlying data was not provided with the article, contrary to the Data Availability statement. In post publication discussions, the corresponding author provided a file described as the data underlying this article.

The corresponding author claimed that the conclusions of this article [1] do not rely on data from the retracted works, and they provided alternative citations. They acknowledged that several statements in the article [1] were not supported by relevant citations and provided alternatives. However, several of these alternative citations could not be located or confirmed.

The *PLOS One* Editors retract this article as the unresolved issues raise concerns about the reliability of the results and conclusions.

NAA and WHA did not agree with retraction. SZO, MAA, RBB, MOA, MAZ, HMA, AFA, SA, SMB, HMA, ODA, BMA, and WAM either did not respond directly or could not be reached.
